# The Pap smear screening as an occasion for smoking cessation and physical activity counselling: effectiveness of the SPRINT randomized controlled trial

**DOI:** 10.1186/1471-2458-12-740

**Published:** 2012-09-05

**Authors:** Giuseppe Gorini, Giulia Carreras, Livia Giordano, Emanuela Anghinoni, Anna Iossa, Alessandro Coppo, Fiorella Talassi, Maurizio Galavotti, Elisabetta Chellini

**Affiliations:** 1 Unit of Environmental and Occupational Epidemiology, Cancer Prevention and Research Institute (ISPO), Via delle Oblate 2, Florence, 50141, Italy; 2 CPO Piedmont, Turin, Italy; 3 Local Health Authority, Mantua, Italy

**Keywords:** Smoking cessation, Counselling, Pap-smear screening

## Abstract

**Background:**

The organized Cervical Cancer Screening Programme (CCSP) in Italy might represent an occasion to deliver smoking cessation (SC) counselling to women attending the Pap test examination. Evidence of effectiveness of physical activity (PA) promotion and intervention in adjunct to SC counselling is not strong.

Objective of the SPRINT trial was to evaluate the effectiveness of a standard SC counselling intervention delivered by trained midwives in the CCSP, and whether the adjunct of a PA counselling to the SC counselling might increase quit rates.

**Methods/Design:**

We undertook a randomized controlled trial of 1,100 women undergoing the Pap examination in the three study centres Florence, Turin, and Mantua: 363 were randomly assigned to the SC counselling arm, 366 to the SC + PA counselling arm, and 371 to the control group. The intervention was a standard brief SC counselling combined with a brief counselling on increasing PA, and was tailored according to the Di Clemente-Prochaska motivational stages of change for SC and/or PA. Primary outcomes were quit rates, improvement in the motivational stages of change for SC, and reduced daily cigarette consumption. Analysis was by intention to treat.

**Results:**

Participants randomized in both intervention arms and in the preparation stage of change for SC doubled their likelihood of quitting at 6-month follow-up in comparison to controls (odds ratio [OR]=2.1, 95% confidence interval [95% CI]:1.0-4.6). Moreover, participants in the intervention arms and in the contemplation stage were more likely to reduce their daily cigarette consumption after the intervention (OR=1.8, 95% CI:1.1-3.0). Our study did not show any effect of PA counselling on various outcomes.

**Conclusions:**

Smoking cessation counselling delivered by midwives to smokers in preparation and contemplation stages of change during the Pap-smear screening was effective and should be recommended, given the high number of women attending the cervical cancer screening programme in Italy. Moreover, the daily number of women invited for the Pap-smear examination should be slightly lowered, in order to let midwives deliver SC counselling to smokers.

**Trial registration:**

Current Controlled Trials ISRCTN52660565

## Background

Smoking is the leading cause of death and of many diseases for both men and women [1]. Since 1980, smoking prevalence in women is stalling in Italy (from 19.2% in 1986 to 17.0% in 2009), in comparison to men [2]. Thus, as the Framework Convention on Tobacco Control suggested [3], there is a need for gender-specific tobacco control strategies. However, these strategies have rarely been developed, except those for pregnant women [4,5].

The organized Cervical Cancer Screening Programme (CCSP) in Italy might represent an occasion to promote healthy lifestyles and deliver smoking cessation counselling to women aged 25–64 years attending the Pap test examination. This primary prevention activity might be easily integrated with the ongoing routine secondary prevention practice. Moreover, smoking is an important co-factor for the development of cervical cancer, being human papilloma-virus (HPV) the principal causal factor [6].

The linkage between smoking cessation and the fear to gain weight is well documented in women. Because calorie reduction may enhance the reinforcing value of smoking cessation, clinical practice guidelines recommended physical activity (PA), rather than diet [4,7]. Physical activity programmes have been proposed as adjuncts to smoking cessation programmes and to relapse prevention programmes [8-11]. Moreover, a minimal intervention strategy aimed at promoting moderate/intensity PA with advice provided by health professionals, based on the trans-theoretical model of behavioural change [12], was effective in producing short-term increases in PA levels [13,14]. Therefore, adding a PA counselling to the standard smoking cessation counselling may improve quit rates among women attending the Pap test examination.

The SPRINT study was designed to evaluate the effectiveness of a standard counselling intervention on smoking cessation delivered by trained midwives in a gender-specific setting (the outpatient cervical cancer screening visits), and whether the adjunct of PA counselling to the standard smoking cessation counselling might increase quit rates among women undergoing the CCSP.

## Methods

**Figure 1:**
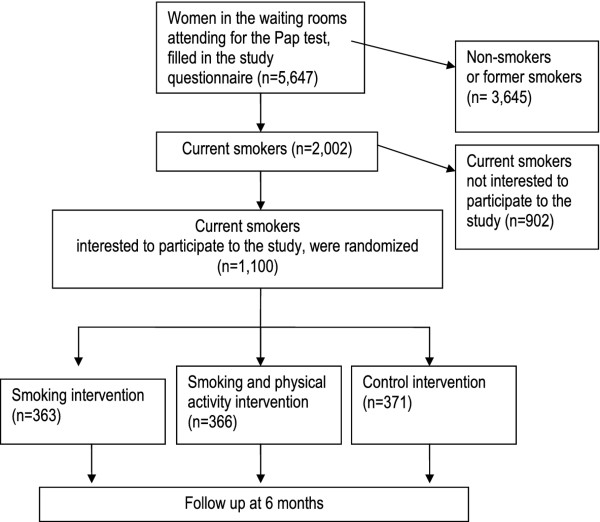
Flow chart of the enrolment of women attending the National Cervical Cancer Screening Programme in Florence, Turin, and Mantua.

Details on the design of the SPRINT study, a three-arm randomized controlled trial, have been already published [15,16], and are briefly summarized here. The study recruited 1,100 smoking women aged 25–64 years attending CCSP consulting rooms: 215 in Florence, 489 in Turin, and 396 in Mantua. Then, 363, 366, and 371 participants were randomly assigned to smoking cessation counselling [SC] arm, the smoking cessation+PA counselling [SC+PA] arm, or to the control arm, respectively (Figure 1)

Women waiting to perform the test were asked to fill in the study questionnaire and participants provided informed consent. Questionnaire regarded demographic characteristics, lifetime and current use of cigarettes, frequency of previous quit attempts, the Fagerström Tolerance Questionnaire (FTQ) [17], intention to quit to assess the Di Clemente-Prochaska stage of change for smoking cessation (precontemplation, contemplation, preparation) [12]. Moreover, 5 stages were distinguished in the questionnaire, following the Di Clemente-Prochaska stages of change for increasing PA (precontemplation, contemplation, preparation, action, or maintenance) [11]. Women who decided to participate handed in the filled questionnaires to the midwives before the Pap-test examination.

A self-help booklet on smoking cessation and increasing PA was provided to all study participants.

Counselling was delivered after the Pap test examination by the same midwives who performed the test. Midwives were trained to deliver counselling for smoking cessation and for increasing PA.

The smoking cessation counselling corresponds to the first two phases of the brief intervention for smoking cessation (“Ask” and “Advice”) [7]. It was tailored according to the Di Clemente-Prochaska stage of change on smoking cessation [12]. If participants were in the precontemplation stage of change for smoking cessation, e.g., women did not even consider quitting, and were randomized to both the SC or the SC + PA arms, midwives’ goal was to move them toward contemplation, and to help women think about quitting. If women were in the contemplation stage of change, e.g., they were thinking of quitting in the next 6 months, midwives’ goal was to tilt the balance toward cessation, and to encourage women to move towards quitting soon. If women were in the preparation stage, i.e., were planning to quit in the next month, midwives’ goal was to motivate to quit, and to help setting a quit-day.

The PA counselling was a brief intervention, designed and validated by psychologists involved in the study [13]. It was a stage-specific counselling, according to the Di Clemente-Prochaska stage assessment on PA [18]. If participants were in the precontemplation stage of change for increasing PA, and were randomized to the SC + PA arm, midwives’ goal was to move them towards contemplation. If women were in the contemplation stage of change for increasing PA, midwives’ goal was to remove their ambivalence on increasing PA. If women were in the preparation stage of change, midwives’ goal was to identify barriers to do more physical exercise. Finally, if women were in the action or maintenance stages of change, e.g., they had begun to do more physical exercise in the last 6 months or for more than 6 months, respectively, midwives reinforced reasons for increasing PA.

A telephone follow-up was scheduled after six months from the intervention, in order to know how many women successfully quitted, and, among those who did not quit, how many improved their motivational stage of change for smoking cessation (e.g., from precontemplation before the intervention to contemplation or preparation after the intervention; from contemplation before the intervention to preparation afterwards), and how many reduced the number of cigarettes smoked per day. Moreover, PA levels after the intervention were assessed during the follow-up interview.

The SPRINT Study was submitted and approved by the Ethics Committee of the Local Health Authority of Florence, Italy (authorization n.143/2009). This trial study is registered, number ISRCTN52660565.

### Analysis

Three main outcomes were analyzed: quitting smoking, improving the motivational stage of change for smoking cessation, and reducing the number of cigarettes per day. Additionally, we analyzed PA outcomes (percentage of women engaged in moderate/intense PA for at least 30 minutes on at least 5 days per week; improvement of their stage of change for increasing PA).

In order to take into account the hierarchical structure of the data, estimates of the intervention effect at six-month follow-up for all centers were obtained with random effects logistic regression models with centre as a random effect, and including age as covariate. The model was not adjusted for baseline characteristics because there were no significant differences among groups at baseline [15]. Analysis was by intention to treat. The software STATA 11 was used for the analyses.

## Results

Among 1,100 participants, 93 were lost to the follow-up (8.5%): 13 participants from Florence (6.0% of Florentine participants), 59 from Turin (12.1%), and 21 from Mantua (5.3%).

**Figure 2:**
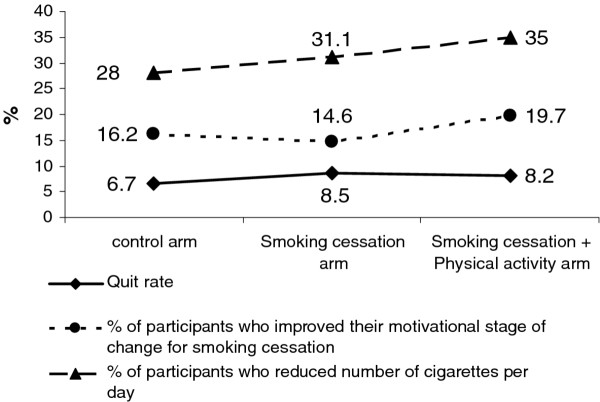
Crude prevalence of study outcomes at 6-month follow-up.

The crude prevalence of smoking outcomes (quit rates; percentage of participants who improved their motivational stage of change for smoking cessation; percentage of participants who reduced their daily cigarette consumption) showed few differences between the two intervention arms. For instance, quit rates were 6.7%, 8.5% and 8.2% for the control arm, SC and SC+PA arms, respectively (Figure 2). A regression model analysis did not show any differences in the smoking outcomes between SC and SC+PA arms (data not shown). Moreover, participants randomized in the SC+PA arm did not report any increase in PA, in terms of percentage of women engaged in moderate/intense PA for at least 30 minutes on at least 5 days per week (odds ratio [OR]=1.10; 95% confidence interval [CI]:0.73-1.68), or in terms of improvement of their stage of change for increasing PA (OR=0.87; 95% CI:0.59-1.26), in comparison to participants in the SC arm.

Since results in the two intervention arms did not differ significantly, we conducted an analysis collapsing the two intervention arms (Table 1). Participants randomized in both the intervention arms and in the preparation stage of change doubled their likelihood of quitting at 6-month follow-up relative to control participants (OR==2.1; 95% CI: 1.0-4.6). On the contrary, participants randomized in the intervention arms and in the precontemplation stage of change at baseline showed a 70% reduction (OR=0.3; 95% CI: 0.1-0.9) in the odds of quitting at 6-month follow-up. Moreover, participants in the intervention arms and in the contemplation stage were more likely to reduce their daily cigarette consumption after the intervention (OR=1.8; 95% CI: 1.1-3.0).

**Table 1:** Adjusted odds ratios (OR) with corresponding 95% confidence intervals (95%CI) of smoking outcomes at the 6-month follow-up (quitting smoking, improving the motivational stage of change for smoking cessation, reducing the number of cigarettes per day) byh motivational stage of change

All centers, by motivational stage of change *		**Quitting smoking**			**Improving the motivational stage of change for smoking cessation**			**Reducing the number of cigarettes per day**		
	**Ntot °**	**N**	**OR §**	**95%CI**	**N**	**OR §**	**95%CI**	**N**	**OR §**	**95%CI**
Precontemplation										
control arm	113	9	1*		28	1*		27	1*	
experimental arms	230	7	0.3	0.1, 0.9	72	1.4	0.8,2.4	68	1.3	0.8,2.2
Contemplation										
control arm	129	6	1*		32	1*		31	1*	
experimental arms	230	15	1.3	0.5, 3.5	53	1.0	0.6, 1.6	86	1.8	1.1, 3.0
Preparation										
control arm	117	9	1*		--	--	--	42	1*	
experimental arms	243	36	2.1	1.0, 4.6	--	--	--	77	0.8	0.5, 1.3
Overall **										
control arm	371	25	1*		60	1*		104	1*	
experimental arms	729	61	1.2	0.8, 2.0	125	1.1	0.8, 1.6	241	1.2	0.9, 1.7

ORs were calculated considering the two collapsed intervention arms with respect to the control arm.

* multilevel analysis.

§ adjusted for age.

° Ntot: participants in the collapsed experimental arms and in the control arm.

** the number of participants reported in the Overall line do not always corresponds to the sum of the number of participants in the three stages of change, because of some missing values.

## Discussion

Our study has shown that smoking cessation counselling delivered by midwives during the Pap-smear screening was effective in increasing 6-month quit rates in women in the preparation motivational stage of change for smoking cessation. Moreover, it was effective in increasing the proportion of women who reduced their daily cigarette consumption among women who did not quit and were in the contemplation motivational stage of change for smoking cessation. On the contrary, our study did not show any effect of PA counselling both on smoking cessation and PA outcomes. Finally, participants in the two intervention arms and in the precontemplation stage of change for smoking cessation recorded significantly lower cessation rates than women of the control group.

Thus, the widespread use of smoking cessation counselling delivered by midwives to smokers in the preparation and in contemplation stages of change for smoking cessation should be recommended during the Pap test. On the contrary, it could be sufficient for smokers in the precontemplation stage to receive a self-help booklet on smoking cessation.

Even though the proportion of women likely to become long-term quitters as a result of a midewife-mediated intervention during the cervical cancer screening is likely to be small (8-9%), however the effect could be important, given the large number of women who could be reached in this setting. In fact, the cervical cancer screening programme in the last years in Italy was attended by about 300,000 current smokers [19].

Our results regarding smoking cessation rates for women in the preparation stage of change were consistent with those recorded in the meta-analysis on nurse-mediated smoking cessation interventions in smokers who were not hospitalized, which showed a significant increase in the success rates in comparison to the control group (OR = 1.84; 95% CI :1.49-2.28) [20].

Even though promoting moderate/intensity PA with advice provided by health professionals, based on the trans-theoretical model of behavioural change, independently from smoking status, was effective in producing short-term increases in number of minutes walked among inactive individuals contemplating changes in their PA levels [13,14], in our study PA counselling did not show any effect on PA and smoking cessation outcomes. A Cochrane review of 13 randomized controlled trials examining PA programmes as a support for smoking cessation concluded that there was limited evidence that it helped [8]. Moreover, a review and meta-analysis on behavioural interventions to promote cessation and prevent weight gain found that combining smoking treatment and behavioural weight control did not produce significantly higher abstinence, than did smoking treatment alone [21]. In a recent trial conducted in Switzerland, participation in a PA programme as an aid for smoking cessation did not significantly increase smoking cessation rates and did not significantly reduce weight gain [22]. On the contrary, a relapse prevention intervention showed that increased moderate to vigorous PA significantly predicted sustained 6-month abstinence [11].

Midwives in all study centers reported that the critical point in delivering smoking cessation counselling in the occasion of the CCSP, was time constraints due to the high number of women invited per day for the Pap test. In order to achieve the goal of including smoking cessation counselling in the daily activity of midwives in the cervical cancer screening setting, focus group were organized and midwives recommended to increase of about 1–2 minutes per woman the expected duration of time visit for doing Pap test.

This study has a number of limitations. First, even though the sample size required 430 participants per arm [15], we recruited only 370 women per arm, because one initially enrolled study center did not conduct the study. Second, all the outcome measures on smoking cessation and PA were self-reported, even though self-reports are considered a low-cost approach to obtaining sufficiently accurate information on tobacco use and PA [23,24]. Third, participants were more likely to be in the preparation stage of change for smoking cessation than non-recruited smokers [15]. This high prevalence of participants in the preparation stage of change could have determined a higher than expected cessation rate in the control group. In fact, cessation probabilities among Italian women aged 30–59 years was about 3.1%-3.7% in 2000–2009 [25], almost the half of that recorded in the control group (about 7%). This could have determined an underestimation of the effect of the intervention. Fourth, we were not able to monitor the different intensity of counselling on PA or smoking cessation according to the motivational stages of change, even though midwives were trained in order to deliver different intensity of counseling.

## Conclusions

Smoking cessation counselling delivered by midwives to smokers in preparation and contemplation stages of change during the Pap-smear screening was effective and should be extended to all the outpatient cervical cancer screening centers, given the high number of women attending the cervical cancer screening programme in Italy. It should also be recommended that the daily number of invited women for the Pap-smear examination should be slightly lowered, in order to let midwives deliver smoking cessation counselling to smokers.

## Competing interest

The authors declare that they have no competing interests.

## Authors’ contribution

GG participated in the design and analysis of the study, and draft the manuscript. GC analyzed the data and contributed to the manuscript draft. LG participated in the design of the study, was responsible of the study in Piedmont, and contributed to the manuscript draft. EA participated in the design of the study, was responsible of the study in Lombardy, and contributed to the manuscript draft. AI participated in the design of the study, was responsible of the study in Tuscany, and contributed to the manuscript draft. EC conceived of the study, participated in the design of the study, coordinated the multicentric study group and contributed to the manuscript draft. All authors read and approved the final manuscript.

## Pre-publication history

The pre-publication history for this paper can be accessed here:

http://www.biomedcentral.com/1471-2458/12/740/prepub
